# Long-term tolerance and cardiac function in breast cancer patients receiving trastuzumab therapy

**DOI:** 10.18632/oncotarget.13726

**Published:** 2016-11-30

**Authors:** Ping Huang, Shujun Dai, Zhimin Ye, Yajuan Liu, Zhanhong Chen, Yabing Zheng, Xiying Shao, Lei Lei, Xiaojia Wang

**Affiliations:** ^1^ Department of Medical Oncology, Zhejiang Cancer Hospital, Hangzhou, China; ^2^ Department of Intense Care Unit, The Second Affiliated Hospital, Zhejiang University School of Medicine, Hangzhou, China; ^3^ Department of Radiation Oncology, Zhejiang Cancer Hospital, Hangzhou, China; ^4^ Department of Tumor Comprehensive Treatment, Hangzhou Cancer Hospital, Hangzhou, China

**Keywords:** breast cancer, Her-2, LVEF, trastuzumab, cardiac event

## Abstract

We examined the long-term clinical tolerance and cardiac safety of trastuzumab treatment in ninety-four female patients diagnosed with breast cancer with human epidermal growth factor receptor 2 (HER-2) overexpression. Electrocardiography (ECG) was monitored throughout trastuzumab treatment, and left ventricular ejection fractions (LVEFs) were estimated using echocardiography prior to treatment with trastuzumab and every 3 months after its first application. The duration of trastuzumab treatments ranged from 3 to 60 months. Declines in LVEF ≥ 15% were seen mainly after 3-15 months of trastuzumab treatment, and LVEF was lowest at 15 months, which coincided with the largest decline in LVEF from baseline. Spearman correlation coefficients indicated that accumulation of anthracycline, the use of cyto/cardioprotective drugs (CPD) and the duration of trastuzumab treatment were all associated with the change of LVEF, and there was a strong correlation between these factors and the change of LVEF (ρ=0.81, ρ=0.734 and ρ=0.777 respectively). These results indicate that significant decreases of LVEF may be seen after 3-15 months of trastuzumab treatment, but that there is a favorable benefit-risk ratio for patients undergoing long-term trastuzumab treatment.

## INTRODUCTION

Treatment with trastuzumab, a humanized monoclonal antibody that binds to the extracellular domain of human epidermal growth factor receptor 2 (HER-2) to target the HER-2 pathway, significantly improves outcomes for women with HER-2-positive breast cancer [1-3]. When administered concurrently or sequentially with systemic chemotherapy, trastuzumab can improve both disease-free survival (DFS) and overall survival (OS) in patients with HER-2-positive breast cancer, though cardiotoxicity remains an important clinical issue, especially for concurrent anthracycline (A) regimens.

Trastuzumab-related cardiotoxicity manifests mainly as a decrease in left ventricular ejection fraction (LVEF) and abnormal cardiac function [2-5]. The short-term cardiac safety surveillance data for trastuzumab has been reported previously, and the overall incidence of cardiac toxicity varies among treatment centers. Cardiotoxicity reportedly occurs in up to 7% of patients when trastuzumab is used as a single agent, but in early clinical trials cardiotoxicity occurred in up to 27% of patients receiving trastuzumab concurrently with anthracycline, and in up to 13% of patients receiving trastuzumab with paclitaxel [6, 7]. On the other hand, long-term tolerance of trastuzumab has rarely been described. In the present study, we investigated the long-term cardiac safety and incidence of cardiotoxicity in patients receiving trastuzumab treatment at our center in southeastern China, and we analyzed the probable factors associated with changes in LVEF.

## RESULTS

### Patient characteristics

A total of 94 patients were enrolled from 2005 through 2014, and a full set of clinical data was obtained from each. The clinical characteristics of the entire study population are shown in Table 1. The median age was 46 years (range, 26-64 years). There were 44 patients who scored a PS of 0, 34 scored a PS of 1, and 16 scored a PS of 2. Over half of the patients (68%) had stage II/III disease. Fifty-six patients underwent adjuvant trastuzumab treatment, and 38 underwent salvage trastuzumab treatment for advanced stage or recurrent disease. About 20 patients suffered from cardiovascular-related risk factors, and 57 underwent left chest irradiation. In addition, 84 (89.4%) patients also received anthracycline-based adjuvant chemotherapy. The mean duration of trastuzumab treatment was 15.73 months (range, 5-60 months). The mean interval between anthracycline and trastuzumab was 4.47 months. About 57 patients accepted cardioprotection drug treatment. Of those, 6 experienced cardio-related symptoms, but none halted the treatment. Abnormal ECG results were seen in 31 patients. The common electrocardiographic abnormalities were sinus tachycardia, sinus bradycardia, and ST-T segment changes. The interval between the initial trastuzumab treatment and electrocardiographic abnormalities was 5.47 ± 3.66 months.

**Table 1:** Characteristics of study population and LVEF level at different time points (n = 94)

Characteristics	N	Characteristics	Mean±SD
PS		Age	46.73±8.91
0	44	Cumulative dose of A (mg/m2)	228.12±174.4
1	34		
2	16	Interval between A and T(M)	4.47±1.45
Stage			
I	10	Interval between R and T(M)	2.44±3.47
II	26		
III	40	Duration of T(M)	15.73±13.18
IV	18	LVEFbaseline	72.13±4.93
Heart disease		LVEF3	69.93±6.36
Yes	0	LVEF6	69.12±5.32
No	94	LVEF9	69.82±6.12
A		LVEF12	69.46±5.54
With	84	LVEF15	68.49±6.39
Without	10	LVEF18	70.21±6.17
CPD		LVEF21	69.25±6.67
With	57	LVEF24	69.39±6.41
Without	37	LVEF27	69.55±4.84
Radiation		LVEF30	69.4±4.77
Left	57	LVEF33	69.64±5.44
Right/without	37	LVEF36	70.19±5.03
ECG		LVEF39	69.89±5.59
N	63	LVEF42	70.69±6.32
AN	31	LVEF45	71.49±7.01
CVD risk		LVEF48	70.36±4.79
With	20	LVEF51	69.23±4.45
Without	74	LVEF54	73.19±3.49
Symptom		LVEF57	70.05±4.18
Yes	6	LVEF60	71.4±5.46
No	88		

PS: Performance Score, A: Anthracycline, CPD: cyto/cardioprotection drugs, ECG: Electrocardiography, N: Normal, AN: Abnormal, CVD: Cardiovascular disease, T: Trastuzumab, LVEF: Left ventricular ejection fraction.

### Monitoring LVEF

**Figure 1:**
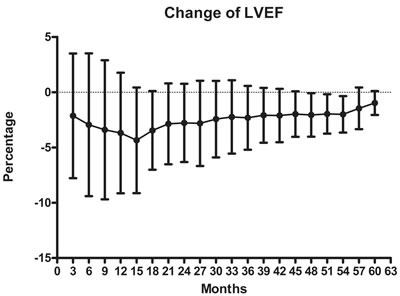
Percent changes in LVEF after 3 months to 60 months of trastuzumab treatment were determined relative to the LVEF prior to treatment (baseline) Symbols depict the mean ± SD. The points on the X-axis represent the changes in LVEF during the period from 3 months to 60 months, respectively.

All the patients underwent echocardiography from baseline to 6 months. The mean baseline LVEF was 72.12% (60.0%-82.43%), mean LVEF at 3 months was 69.93% (53.7%-82.8%), and mean LVEF at 6 months was 69.12% (58.86%-79.7%). The values of LVEF at every time point are listed in Table 1. Changes in LVEF were calculated as the LVEFratio and are illustrated in Figure 1. Note that the magnitudes of the decreases in LVEF increased during the period from 3 months to 15 months, and then steadily decreased 15 months. LVEF was lowest at 15 months. In Table 2, the magnitudes of the changes are divided into 5 ranks, and the numbers of cases in each rank are listed.

**Table 2:** Changes of LVEF were divided into 5 ranks and the number of cases was listed

LVEFratio	3M(n=94)	6M(n=94)	9M(n=90)	12M(n=84)	15M(n=73)	18M(n=62)	~24M(n=56)	~36M(n=44)	~48M(n=30)	~60M(n=21)
>0	24(25.5%)	18(19.11%)	16(17.8%)	18(21.4%)	14(19.8%)	19(30.6%)	18(32.1%)	17(38.6%)	14(46.7%)	10(47.6%)
-5%~0	36(38.3%)	40(42.6%)	35(38.9%)	32(38.1%)	24(32.9%)	18(29%)	19(33.9%)	17(38.6%)	13(43.3%)	9(42.9%)
-10%~-5%	22(23.4%)	25(26.6%)	27(30%)	25(29.8%)	25(34.2%)	20(32.3%)	16(28.6%)	10(22.8%)	2(6.7%)	2(9.5%)
-15%~-10%	9(9.6%)	7(7.4%)	8(8.9%)	6(7.1%)	6(8.2%)	3(4.8%)	2(3.6%)	0(0)	1(3.3%)	0(0)
≤-16%	3(3.2%)	4(4.3%)	4(4.4%)	3(3.6%)	4(5.5%)	2(3.2%)	1(1.8%)	0(0)	0(0)	0(0)

### Correlation between main clinical factors and changes in LVEF

**Figure 2:**
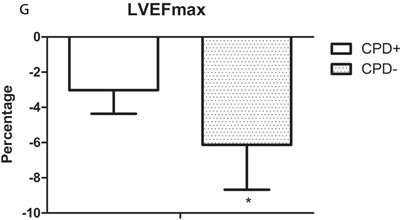
The correlations between several clinical factors and trastuzumab-induced changes of LVEF were analyzed The histograms depict the maximal shifts in LVEF (LVEFmax). Shown is the mean ± SD. **A.** Patients who received anthracycline (A+) *vs*. those who did not (A-) (*P* = 0.068). **B.** Patients who received a cumulative anthracycline dose of < 300 mg/m^2^ *vs*. those who received > 300 mg/m^2^ (*P < 0.05). **C.** Patients with cardiovascular diseases risk factors (CVRF+) *vs*. those without risk factors (CVRF-). **D.** Patients who received left chest radiation (LR) *vs*. those who received right chest radiation/no radiation (RR/R-). **E.** Patients receiving trastuzumab (T) for ≤15 months *vs*. those receiving T > 15 months (*P < 0.05). **F.** Patients were divided into 3 groups based on PS scoring. **G.** Patients who received cyto/cardioprotective drugs (CPD+) *vs*. those who did not (CPD-) (*P < 0.05).

To assess the correlation between the main clinical factors and changes in LVEF, the maximal shift in LVEF in each patient (LVEFmax) was calculated, after which the LVEFmax values were grouped according to clinical factors, including PS score, anthracycline (+/-), chest radiation, cardiovascular risk factors (+/-), cyto/cardioprotective drugs (+/-), and duration of trastuzumab. The results summarized in Figure 2 indicate that cumulative anthracycline dose (A), cyto/cardioprotective drugs (CPD) and duration of trastuzumab are associated with the change of LVEF (*P* < 0.05). A larger decrease in LVEF during the course of treatment occurred mainly in the patients with a cumulative dose of anthracycline > 300 mg/m^2^, without cyto/cardioprotective drugs, and a trastuzumab treatment duration of 15 months. Spearman correlation coefficients were calculated to analyze the correlation between the aforementioned factors and LVEFmax. Among these factors, use of cyto/cardioprotective drugs, cumulative dose of anthracycline and duration of trastuzumab administration all strongly correlated with LVEFmax (*ρ* = 0.81, *ρ* = 0.734 and *ρ* = 0.777). These results are indicative of the utility of cyto/cardioprotection drugs for preserving cardiac function during trastuzumab treatment.

## DISCUSSION

The cardiotoxicity of trastuzumab continues to be a focus of attention for clinical oncologists. In the present study, the duration of trastuzumab treatment ranged from 3 months to 60 months, and among 94 patients, 21 underwent trastuzumab treatment for > 48 months. To the best of our knowledge, little has been reported on the cardiotoxicity of long-term trastuzumab in patients undergoing treatment in China. The results showed that there is a low incidence of symptomatic cardiac events after trastuzumab treatment, with or without anthracycline. After a prolonged period, we observed a few cases in which LVEF declined 16%, but such late onset cardiac dysfunction with trastuzumab was rare, and long-term use of trastuzumab appears relatively safe (Table 2). This finding is fairly consistent with other pivotal adjuvant trastuzumab trials [6, 8]. It is worth mentioning that the interval during which declines in LVEF were greatest ranged from 3 months to 15 months of trastuzumab treatment, with a trough at the 15-month point (Figure 1), which is distinct from other studies.

To gain a better understanding of the factors affecting the change in LVEF, several probable clinical factors were analyzed, and use of cyto/cardioprotective drugs, cumulative dose of anthracycline and duration of trastuzumab administration all correlated to the change of LVEF (Figure 2B, 2E, 2G). The results further showed that the cardiotoxic effect of trastuzumab may be alleviated by cyto/cardioprotective drugs. It was also previously reported that patients may benefit from cyto/cardioprotective drugs during chemotherapy [9]. Shenmai injection, a popular herbal preparation in China, is widely used for maintenance of myocardial function in patients with coronary artery disease [10, 11]; however, there have been no studies investigating the efficacy of Shenmai injection in patients receiving trastuzumab. On the other hand, protocols that include trastuzumab in aggressive radiochemotherapy are reportedly feasible when supported with amifostine [12, 13]. In addition, levocarnitine, a naturally occurring essential co-factor in fatty acid metabolism, exerts a protective effect in patients with ischemic heart disease, which is correlated a reduction in oxidative stress injury [14]. The efficacy of levocarnitine in patients receiving trastuzumab remains unknown. In the present study, the magnitude in the decline in LVEF was significantly smaller in patients receiving cyto/cardioprotective drugs than in those without these drugs. We observed that patients receiving anthracycline treatment with a cumulative dose > 300 mg/m^2^ were most likely to suffer a decrease in LVEF. Retrospective analyses from clinical trials in adults suggest that the incidence of cardiac function damage due to doxorubicin is 1.7% at a cumulative dose of 300 mg/m^2^ [15], which is similar our finding. Moreover, analysis of pooled data from six trials of treatments for metastatic breast cancer showed that 11.6% of patients treated with trastuzumab after prior anthracyclines experienced a decline of LVEF ≥15 points to a level of < 50% [16]. The ratio was higher than in our study, which may be partially attributable to differences in the disease stages of the patients and the doses of anthracycline.

Previous studies suggest that patients with multiple risk factors, such as hypertension, obesity, dyslipidemia, and metabolic syndrome, are more likely to experience cardiotoxicity during trastuzumab treatment [17, 18]. In the present study, however, cardiovascular risk factors did not obviously influence the change in LVEF during the trastuzumab treatment. The widely accepted traditional view is that a cardiovascular response is one of the main complications in breast cancer patients who undergo radiotherapy. This means that radiotherapy, especially left chest radiotherapy, may aggravate the injury to the heart from trastuzumab. However, we observed no significant difference in the change of LVEF between left/right radiation or no radiation. This is consistent with the observations reported by Koukourakis [13], which revealed that inclusion of trastuzumab during radiotherapy for breast cancer did not increase systemic radiation toxicity.

In conclusion, a decrease in LVEF may be observed during the first year in patients receiving trastuzumab treatment, but a progressive decrease in LVEF seldom occurred, even after prolonged treatment intervals. Instead, trastuzumab was well tolerated by HER-2-positive breast cancer patients, whether it was administered sequentially with anthracycline or concurrently/sequentially with radiotherapy. Nonetheless, patients may benefit from the use of cyto/cardioprotective drugs.

## PATIENTS AND METHODS

### General clinical data

From June 2008 to June 2015, a total of 94 female patients diagnosed with breast cancer with overexpression of HER-2 underwent trastuzumab treatment and were enrolled in the study. The study was approved by the regional ethics committee of our hospital, and all patients signed informed consent forms before trastuzumab treatment. All participants met the following criteria: (1) Her-2 overexpression based on the standard of 3+ using IHC or fluorescence *in situ* hybridization [FISH] Ratio > 2.0; (2) ECOG PS ≤ 2; (3) no concomitant congenital heart disease or myocardial infarction; (4) baseline LVEF > 50%; (5) good compliance. All patients’ clinical characteristics are listed in Table 1. Among these factors, cardiovascular disease risk factors were according to the CDC/ACSM guidelines, mainly including hypertension, high BMI, dyslipidemia, and metabolic syndrome.

### Treatment protocols

All participants received trastuzumab according to the dosing regimen recommended by the manufacturer (initial dose, 8 mg/kg; followed by doses of 6 mg/kg every 3 weeks), and each administration was completed in 90 min. Additional chemotherapy or radiotherapy may have been concurrent with or sequential to trastuzumab. It was recommended but not mandated that patients received cyto/cardioprotection drugs during the course of trastuzumab treatment. These included Shenmai injection, amifostine and levocarnitine.

### Recording of symptoms and monitoring of ECG

All participants received ECG examinations before and 1 month after trastuzumab treatment, and heart-related symptoms such as chest distress, dyspnea and palpitation were recorded. If a patient had symptoms of disease, ECG examinations were given each month.

### Evaluation of cardiac function and measures of treatment

Echocardiographic examinations were given to all participants before beginning trastuzumab treatment (baseline) and every 3 months during the treatment in order to measure LVEFs. Changes in LVEF at all time points were determined relative to the LVEF measured at baseline (LVEFratio) and defined as, LVEFratio = (LVEFother points - LVEFbaseline) / LVEFbaseline. If the LVEFratio was ≥16% or LVEF was < 50%, trastuzumab treatment was halted temporarily for more than 4 weeks, and echocardiography was performed every 4 weeks. Trastuzumab treatment was continued if the LVEF recovered to the normal level or the absolute decline was < 15% in 4 to 8 weeks. If there was insufficient recovery after > 8 weeks, trastuzumab administration was halted permanently.

To evaluate the correlation between the main clinical factors and the change of LVEF, the maximal shift in LVEF in each case during the course of trastuzumab treatment (LVEFmax) was calculated as, LVEFmax = (LVEFlowest - LVEFbaseline) / LVEFbaseline.

### Data collection

Patient demographics and baseline characteristics, including cardiovascular disease risk factors, treatment with radiotherapy, interval between anthracycline (A) and trastuzumab, duration of trastuzumab, LVEF level at every time point, and ECG were obtained from existing data.

### Statistical analysis

All statistical analyses were carried out using SPSS 16.0 software. Descriptive statistics were produced for continuous variables. Results are presented as the mean ± SD for continuous variables. The significance of continuous variables was assessed using analysis of variance (ANOVA). The chi-square test was used to determine the significance of rate variables. A Spearman correlation coefficient was used to analyze the correlation between main clinical factors and changes in LVEF. Values of *P* < 0.05 were considered significant.
